# Supplementary material for: RNAseq analysis of oocyte maturation from the germinal vesicle stage to metaphase II in pig and human
Source: PLoS One. 2024 Aug 9;19(8):e0305893. doi: 10.1371/journal.pone.0305893 (PMC11315340; doi:10.1371/journal.pone.0305893)
Supplement: S1 Fig — (PDF) [file pone.0305893.s023.pdf]

Supplementary information

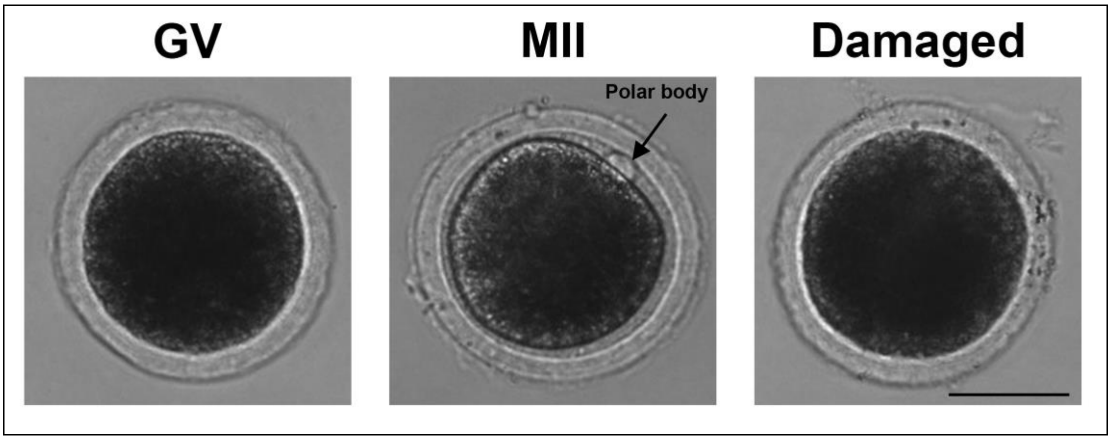

S1 Fig. Morphology of pig oocytes collected for RNAseq. Bright filed images of oocytes at three major developmental stages, GV, MII with polar body, and oocytes without visible polar body ('damaged') after *in vitro* incubation. Scale bar: 80 μm.
