# Supplementary material for: RNAseq analysis of oocyte maturation from the germinal vesicle stage to metaphase II in pig and human
Source: PLoS One. 2024 Aug 9;19(8):e0305893. doi: 10.1371/journal.pone.0305893 (PMC11315340; doi:10.1371/journal.pone.0305893)
Supplement: S2 Fig — (PDF) [file pone.0305893.s024.pdf]

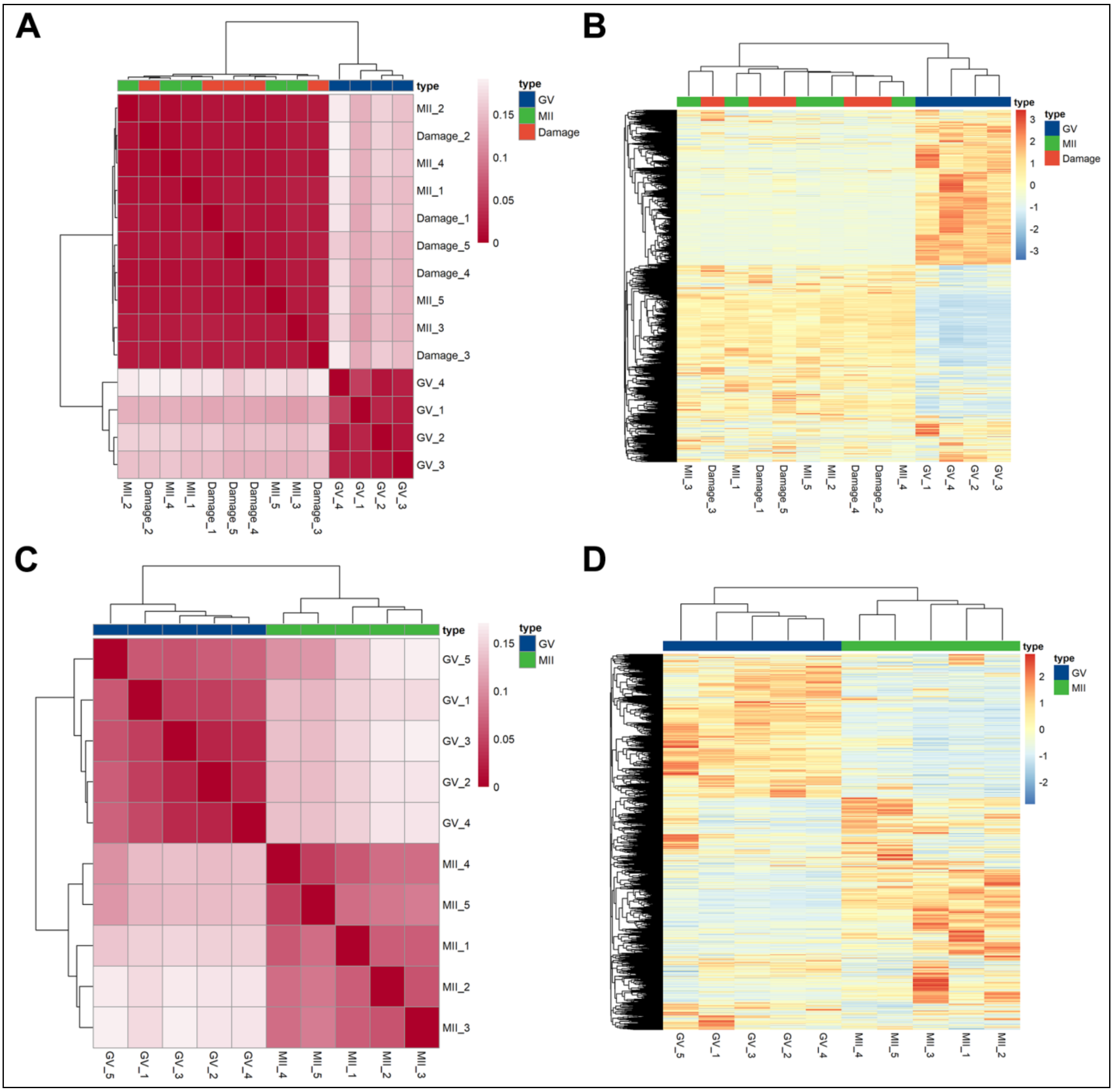

S2 Fig. Heatmap highlighting the distance and gene expression among pig or human oocytes at different developmental stages. A distance matrix generated by sample-by-sample correlation (Pearson correlation) highlighting transcriptome relationships among oocytes at three different stages (GV, MII and damaged) in pig dataset (A) and at two different stages (GV and MII) in human dataset (B). Colour gradient ranges from dark red (minimum distance) to light red. A heatmap showing 12,962 genes expression in pig dataset (C) and 18,998 genes expression in human dataset (D). Colour gradient ranges from blue (low expression) to red (high expression).
