# Supplementary material for: RNAseq analysis of oocyte maturation from the germinal vesicle stage to metaphase II in pig and human
Source: PLoS One. 2024 Aug 9;19(8):e0305893. doi: 10.1371/journal.pone.0305893 (PMC11315340; doi:10.1371/journal.pone.0305893)
Supplement: S4 Fig — (PDF) [file pone.0305893.s026.pdf]

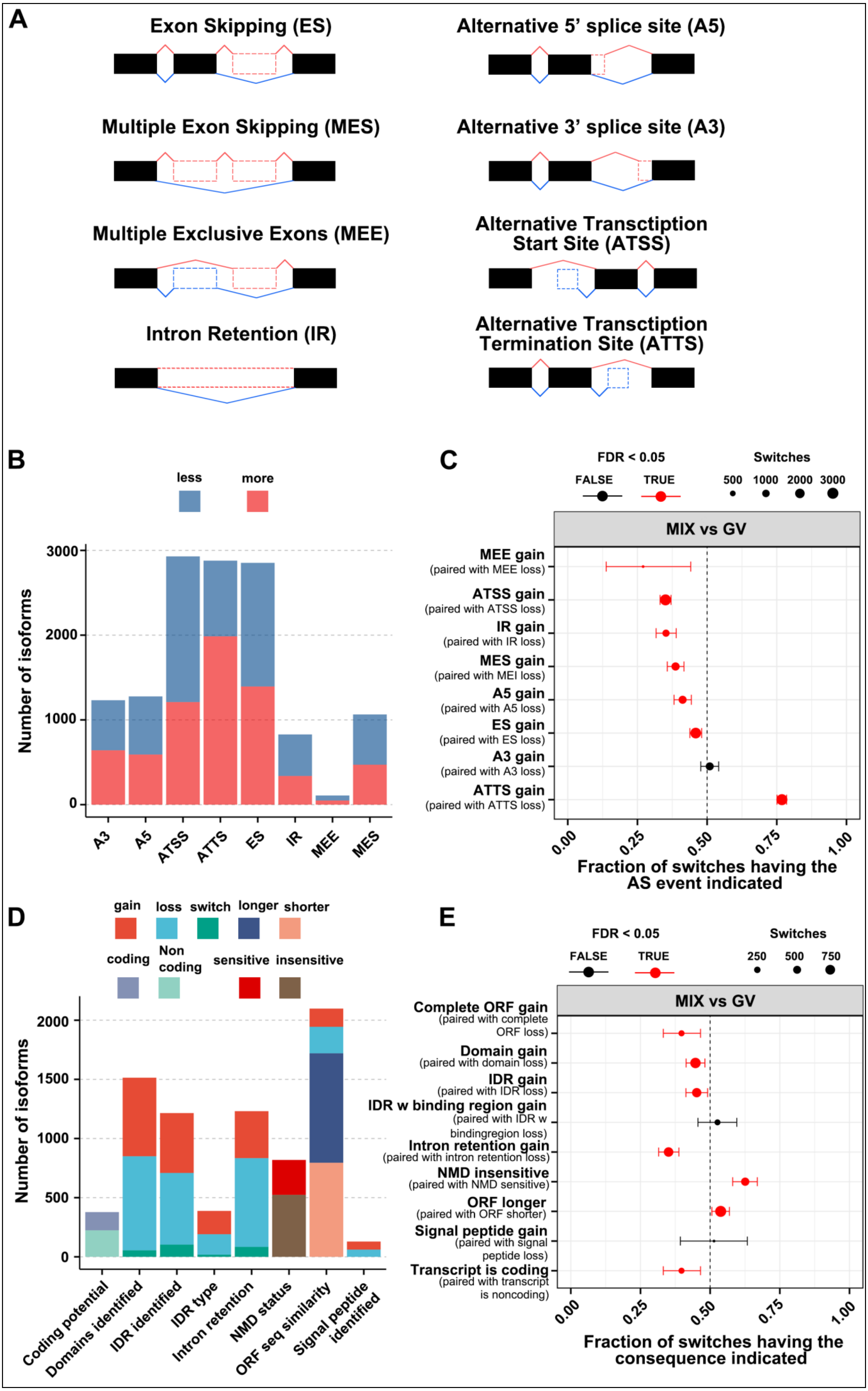

A
Exon Skipping (ES)
Alternative 5' splice site (A5)
Multiple Exon Skipping (MES)
Alternative 3' splice site (A3)
Multiple Exclusive Exons (MEE)
Alternative Transctiption Start Site (ATSS)
Intron Retention (IR)
Alternative Transctiption Termination Site (ATTS)
B
less
more
Number of isoforms
3000
2000
1000
0
A3
A5
ATSS
ATTS
ES
IR
MEE
MES
C
FDR < 0.05
FALSE
TRUE
Switches
500
1000
2000
3000
MIX vs GV
MEE gain
(paired with MEE loss)
ATSS gain
(paired with ATSS loss)
IR gain
(paired with IR loss)
MES gain
(paired with MEI loss)
A5 gain
(paired with A5 loss)
ES gain
(paired with ES loss)
A3 gain
(paired with A3 loss)
ATTS gain
(paired with ATTS loss)
0.00
0.25
0.50
0.75
1.00
Fraction of switches having the AS event indicated
D
gain
loss
switch
longer
shorter
coding
Non coding
sensitive
insensitive
Number of isoforms
2000
1500
1000
500
0
Coding potential
Domains identified
IDR identified
IDR type
Intron retention
NMD status
ORF seq similarity
Signal peptide identified
E
FDR < 0.05
FALSE
TRUE
Switches
250
500
750
MIX vs GV
Complete ORF gain
(paired with complete ORF loss)
Domain gain
(paired with domain loss)
IDR gain
(paired with IDR loss)
IDR w binding region gain
(paired with IDR w bindingregion loss)
Intron retention gain
(paired with intron retention loss)
NMD insensitive
(paired with NMD sensitive)
ORF longer
(paired with ORF shorter)
Signal peptide gain
(paired with signal peptide loss)
Transcript is coding
(paired with transcript is noncoding)
0.00
0.25
0.50
0.75
1.00
Fraction of switches having the consequence indicated

S4 Fig. Alternative splicing mechanisms associated with isoform switches and consequences for protein expression in pig oocytes. (A) Schematic representation of splicing events that can lead to gene isoform switches. (B) Quantification of the number of isoforms showing more (positive difference in isoform fraction (dIF)) and less (negative dIF) splicing events in 'MIX' compared to GV. (C) Splicing events enrichment involved in isoform switches. The x-axis of the plot shows the fraction of switches connected with the indicated splicing event, where < 0.5 means depleted while > 0.5 means enriched in 'MIX'. (D) Quantification of the number of isoforms showing (i) gain or loss of coding potential, domains/signal peptides identified, intrinsically disordered regions (IDR), intron retention, open-reading frame (ORF) sequencing similarity; (ii) switch (simultaneous gain and loss) of domains identified or IDR; (iii) sensitive or insensitive to nonsense-mediated decay (NMD); and (iv) longer or shorter ORF sequencing similarity. (E) The enrichment of functional consequences for protein expression resulting from isoform switching events. The x-axis of the plot shows the fraction of switches having the indicated consequence, where < 0.5 means depleted while > 0.5 means enriched in MIX.
