# Supplementary material for: RNAseq analysis of oocyte maturation from the germinal vesicle stage to metaphase II in pig and human
Source: PLoS One. 2024 Aug 9;19(8):e0305893. doi: 10.1371/journal.pone.0305893 (PMC11315340; doi:10.1371/journal.pone.0305893)
Supplement: S5 Fig — (PDF) [file pone.0305893.s027.pdf]

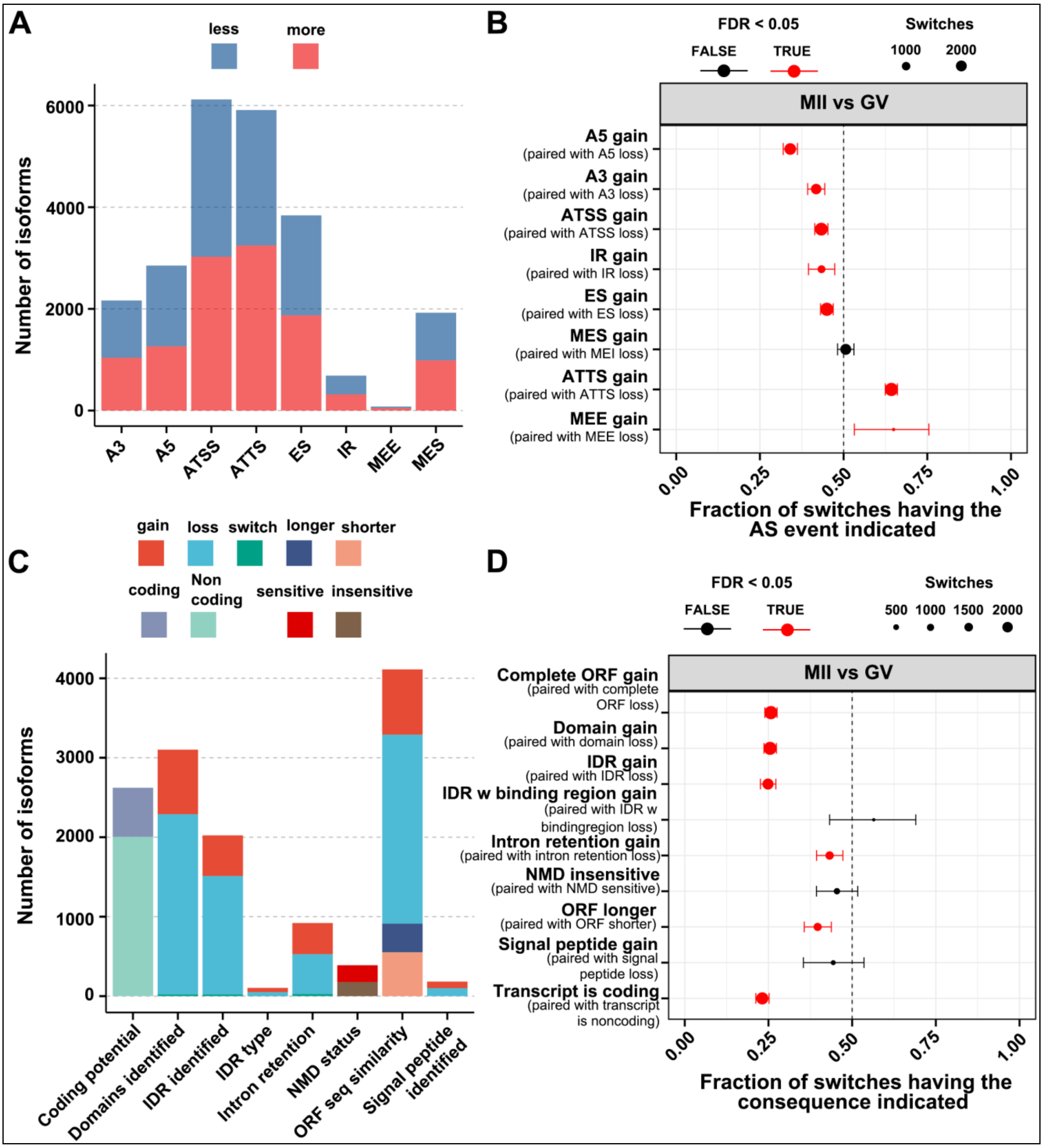

S5 Fig. Alternative splicing mechanisms associated with isoform switches and consequences for protein expression in human oocytes. (A) Quantification of the number of isoforms showing more (positive difference in isoform fraction (dIF)) and less (negative dIF) splicing events in MII compared to GV. (B) Splicing events enrichment involved in isoform switches. The x-axis of the plot shows the fraction of switches connected with the indicated splicing event, where < 0.5 means depleted while > 0.5 means enriched in MII. (C) Quantification of the number of isoforms showing (i) gain or loss of coding potential, domains/signal peptides identified, intrinsically disordered regions (IDR), intron retention, open-reading frame (ORF) sequencing similarity; (ii) switch (simultaneous gain and loss) of domains identified or IDR; (iii) sensitive or insensitive to nonsense-mediated decay (NMD); and

(iv) longer or shorter ORF sequencing similarity. (D) The enrichment of functional consequences for protein expression resulting from isoform switching events. The x-axis of the plot shows the fraction of switches having the indicated consequence, where < 0.5 means depleted while > 0.5 means enriched in MII.
