# Supplementary material for: RNAseq analysis of oocyte maturation from the germinal vesicle stage to metaphase II in pig and human
Source: PLoS One. 2024 Aug 9;19(8):e0305893. doi: 10.1371/journal.pone.0305893 (PMC11315340; doi:10.1371/journal.pone.0305893)
Supplement: S3 Fig — (PDF) [file pone.0305893.s025.pdf]

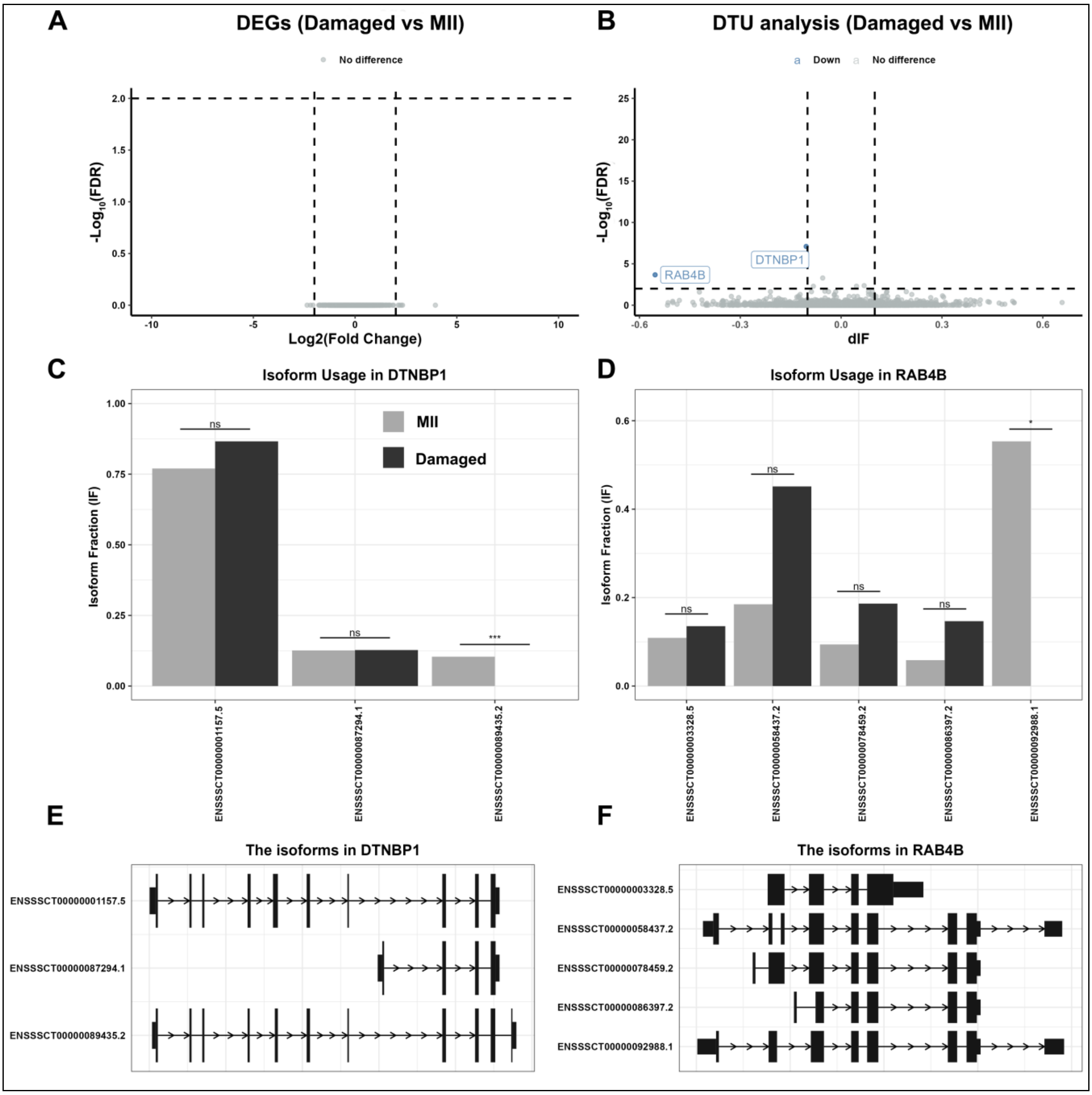

S3 Fig. DGE and DTU analysis comparing pig MII oocytes with damaged oocytes. (A) Volcano plot showing no DE genes (FDR < 0.01 and |fold change| > 4) by DGE analysis comparing pig MII versus damaged oocytes. (B) Two differential transcript isoforms (|dIF| > 0.1 and FDR < 0.01) from two individual genes (*RAB4B* and *DTNBP1*) involved in isoform switching were identified by DTU analysis comparing pig MII versus damage oocytes. (C) Quantification of the isoform fraction in MII and damaged MII oocytes for the three *DTNBP1* isoforms. (D) Quantification of the isoform fraction in MII and damage MII oocytes for the five *RAB4B* isoforms. (E) Schematic representations of exon–intron maps of the three *DTNBP1* isoforms. (F) Schematic representations of exon–intron maps of the five *RAB4B* isoforms.
